# Sex-Dependent Modulation of Acute Stress Reactivity After Early Life Stress in Mice: Relevance of Mineralocorticoid Receptor Expression

**DOI:** 10.3389/fnbeh.2019.00181

**Published:** 2019-08-07

**Authors:** Valeria Bonapersona, Ruth Damsteegt, Mirjam L. Adams, Lisa T. C. M. van Weert, Onno C. Meijer, Marian Joëls, Ratna Angela Sarabdjitsingh

**Affiliations:** ^1^Department of Translational Neuroscience, UMC Utrecht Brain Center, Utrecht University, Utrecht, Netherlands; ^2^Department of Internal Medicine, Leiden University Medical Center, Division of Endocrinology, Leiden, Netherlands; ^3^University Medical Center Groningen, University of Groningen, Groningen, Netherlands

**Keywords:** mineralocorticoid receptor, early life stress (ELS), HPA axis, neuroendocrine, behavior, corticosterone, sex, nuclear receptors

## Abstract

Early life stress (ELS) is considered a major risk factor for developing psychopathology. Increasing evidence points towards sex-dependent dysregulation of the hypothalamic-pituitary-adrenal (HPA) axis as a contributing mechanism. Additionally, clinical studies suggest that the mineralocorticoid receptor (MR) may further confer genetic vulnerability/resilience on a background of ELS. The link between ELS, sex and the HPA axis and how this interacts with MR genotype is understudied, yet important to understand vulnerability/resilience to stress. We used the early life-limited nesting and bedding model to test the effect of ELS on HPA properties in adult female and male mice carrying a forebrain-specific heterozygous knockout for MR. Basal HPA axis activity was measured by circadian peak and nadir corticosterone levels, in addition to body weight and weight of stress-sensitive tissues. HPA axis reactivity was assessed by mapping corticosterone levels after 10 min immobilization. Additionally, we measured the effects of ELS on steroid receptor [MR and glucocorticoid receptor (GR)] levels in the dorsal hippocampus and medial prefrontal cortex (mPFC) with western blot. Finally, behavioral reactivity towards a novel environment was measured as a proxy for anxiety-like behavior. Results show that HPA axis activity under rest conditions was not affected by ELS. HPA axis reactivity after immobilization was decreased by ELS in females and increased, at trend-level in males. This effect in females was further exacerbated by low expression of the MR. We also observed a sex*ELS interaction regarding MR and GR expression in the dorsal hippocampus, with a significant upregulation of MR in males only. The sex-dependent interaction with ELS was not reflected in the behavioral response to novel environment and time spent in a sheltered compartment. We did find increased locomotor activity in all groups after a history of ELS, which attenuated after 4 h in males but not females regardless of condition. Our findings support that ELS alters HPA axis functioning sex-dependently. Genetic predisposition to low MR function may render females more susceptible to the harmful effect of ELS whereas in males low MR function promotes resilience. We propose that this model may be a useful tool to investigate the underlying mechanisms of sex-dependent and genetic vulnerability/resilience to stress-related psychopathology.

## Introduction

Early life is a sensitive developmental period, during which stress experienced early in life (early life stress, ELS), may induce long-lasting consequences for the ability to deal with challenging situations later in life (Heim et al., 2008; Strüber et al., 2014). ELS is known to affect both basal and stress-induced hypothalamus-pituitary-adrenal (HPA) axis activity (van Bodegom et al., 2017). This most likely occurs through gradual but persistent changes in HPA axis circuitry and the wiring of other neural networks involved in cognitive and emotional functioning (Andersen, 2003; Lupien et al., 2009).

Dysregulation of the HPA axis is considered to be an important risk factor for mental illnesses such as major depression and anxiety disorders (de Kloet et al., 1998; Varghese and Brown, 2001; Young et al., 2004; Pariante and Lightman, 2008; Moreno-Peral et al., 2014). Ample evidence associates changes in HPA axis function with depression or anxiety disorders, both with respect to circadian rhythmicity, response to the dexamethasone-CRH test or cortisol responses evoked by psychosocial stress (Künzel et al., 2003; Pariante and Lightman, 2008; Keller et al., 2017; Zorn et al., 2017). This body of literature includes observations in high-risk proband of women with depression, who do not (yet) show any symptoms of depression themselves (Modell et al., 2005), suggesting a causative role of HPA axis disturbances in the precipitation of depressive symptoms. Interestingly, a recent meta-analysis revealed that the association between stress-induced cortisol responses and stress-related psychopathology is sex-dependent: women with depression or anxiety disorders overall showed a blunted response to psychosocial stress (i.e., the Trier social stress test, TSST), whereas men showed the opposite (Zorn et al., 2017). Of note, women exposed to ELS also showed a blunted response to the TSST (Carpenter et al., 2011), while DeSantis et al. (2011) demonstrated a sex-dependent interaction between ELS and the response to CRH. All in all, this suggests that ELS in a sex-dependent manner may dysregulate HPA axis reactivity, which adds to the vulnerability to depression.

The above-mentioned evidence does not take into account that not every individual is equally sensitive to ELS; the latter is also determined by an individual’s genetic background. This is particularly relevant for genes involved in HPA axis reactivity. One such protein is the mineralocorticoid receptor (MR), encoded by the gene *Nr3c2*. The MR binds corticosteroids (CORT) in the brain and is highly expressed in some limbic areas, e.g., in the hippocampus and medial prefrontal cortex (mPFC; Reul and de Kloet, 1985; Joëls et al., 2008). The MR is thought to exist in two forms: nuclear and membrane-associated; these can be functionally differentiated by their affinity for CORT (Kretz et al., 2001; Karst et al., 2005). The nuclear MR has a high affinity and is substantially activated even at the nadir of the circadian cycle. It mediates genomic and slow effects, which are involved in setting the threshold for HPA axis reactivity (Joëls et al., 2008). Conversely, the membrane-associated MR has a lower affinity for CORT, presumably lending it a role in the immediate (cognitive) response to stress (Joëls et al., 2015; Vogel et al., 2016). In order to adequately regulate the response to stress, MR works in close conjunction with the glucocorticoid receptor (GR; Reul and de Kloet, 1985; de Kloet et al., 2005).

Several MR single nucleotide variants (SNPs) have been described, each contributing to HPA axis regulation and the response to stress (van Leeuwen et al., 2010, 2011; DeRijk et al., 2011; Gerritsen et al., 2017). Two SNPs, rs2070951 (MR-2C/G; a C/G SNP) and rs5522 (MRI180V; an A/G SNP), were shown to be inherited in three common haplotypes, i.e., haplotype 1 (GA); haplotype 2 (CA) and haplotype 3 (CG; van Leeuwen et al., 2010). *In vitro*, haplotype 1 and 3 resulted in lower MR expression and transactivation capacity than haplotype 2 (van Leeuwen et al., 2011). MR expression and haplotypes are considered to be important in the vulnerability to stress-related psychopathology (DeRijk et al., 2011; ter Heegde et al., 2015; Joëls and de Kloet, 2017; Wingenfeld and Otte, 2019), in a sex-dependent manner. Thus, women carrying haplotype 1 or 3, compared to those homozygous for haplotype 2, showed lower HPA and autonomic responses after experiencing a psychosocial stressor (van Leeuwen et al., 2011), higher stress scores (van Leeuwen et al., 2011), lower levels of optimism and higher levels of hopelessness (Klok et al., 2011; Hamstra et al., 2017) as well as a higher risk for depression (Klok et al., 2011). Of relevance, an ELS-by-sex-by-MR haplotype interaction was observed in a large sample of healthy individuals and a mixed healthy and clinical cohort. Thus, women exposed to ELS that carry haplotype 1 or 3 showed higher scores on a depression scale than women homozygous for haplotype 2, while this was not observed (or even the opposite) in males (Vinkers et al., 2015). Overall, this suggests that low MR function in females may exacerbate the influence of ELS on HPA axis reactivity and depression, while it might be protective in males.

This notion is not easy to test in a controlled and prospective manner in human cohorts. Therefore, we reverted to a mouse model to test the following hypotheses: first, ELS affects the HPA axis response to the acute stress of adult female mice differently than the response of males. Based on the human literature, we expect that adult female mice exposed to ELS are hypo-responsive to stress, while males exposed to ELS are hyper-responsive. Second, MR expression interacts with ELS effects in a sex-dependent manner; more specifically, we expect that down-regulation of MR causes exacerbation of ELS effects in females, while it serves a protective role in males.

To provoke ELS in a controlled manner, dams (and their litter) were housed in limited nesting and bedding conditions between postnatal day (P) 2 and 9 (Rice et al., 2008). We mimicked the conditions of MR haplotypes 1 and 3 (compared to haplotype 2) by decreasing MR expression, using a forebrain-specific heterozygous MR knock-out mouse, which includes the dorsal hippocampus and mPFC (Berger et al., 2006). In the adult male and female mice, we investigated several indices of HPA axis function, i.e., body weight and weight of stress-sensitive tissues including adrenals and thymus; circadian and stress-induced variations in CORT level; and MR and GR expression in the dorsal hippocampus and mPFC. In addition, we measured behavioral reactivity towards novelty and time spent in covered (as opposed to open) spaces, as a proxy for anxiety-like behavior.

## Materials and Methods

### Animals and Housing Conditions

The current study was approved by the Animal Ethical Committee from Utrecht University, Netherlands. Every effort was taken to minimize animal suffering in accordance with the FELASA guidelines and the Dutch regulation for housing and care of laboratory animals (January 30th 2001/GZB/VVB 2148400). Experiments were performed blindly; animal distribution across experiments, as well as experimental order, was randomized. Experimental cages were randomly placed on the housing racks in stables with temperature (22 ± 2°C) and humidity (~64%) control with reversed day-night cycle (light on 20:00–08:00). Standard chow pellets (Special Diet Services, UK) and tap water were provided *ad libitum*. Where possible, animals were socially housed with same sex and experimental littermates. Throughout all procedures, experimental manipulation and disturbances were kept to a minimum to avoid handling effects.

### Breeding of Experimental Animals

For a detailed description of the mouse lines and breeding schedules, see Berger et al. (2006) and Knop et al. (2019). All experimental and breeding mouse lines were routinely maintained in the animal facility of the Department of Translational Neuroscience, UMC Utrecht Brain Center, Utrecht University to uphold a stable environment and prevent any stress that may otherwise be caused by transportation. Full forebrain-specific MR-knockout breeding males (MR_CamKCre/wt_; MR_flox/flox_) were generated by crossing forebrain-specific CAMKII transgenic mice (purchased from the EMMA mouse repository) with MR_flox/flox_ female mice (loxP site flanked at MR exon 3; kindly provided by Dr. Stefan Berger).

For translational purposes, we modeled low rather than completely absent MR expression and therefore chose to study MR heterozygous knockout mice (MR_CamKCre/wt_; MR_flox/wt_) as an experimental model. To obtain this genotype in the offspring, one full knockout breeding male was paired with two in-house bred naive wildtype C57Bl/6JOlaHsd dams (10–12 weeks old; regularly purchased from Harlan, France); we preferred these wildtype females, to control for possible genetic differences in maternal care. The offspring consisted of heterozygous mice (MR_CamKCre/wt_; MR_flox/wt_) and litter-mate controls (MR_wt/wt_; MR_flox/wt_), later referred to as MR_CamKCre/wt_ and MR_flox/wt_ for simplicity, respectively.

### Genotyping

Genotyping was routinely performed on material obtained from ear-punching at weaning (PND21). DNA was isolated by degrading the material with 0.1 mg/ml Proteinase K in lysis buffer containing 1 M Tris pH 8.0, 0.5 M EDTA pH 8.0, 10% SDS, 5 M NaCl, and distilled water. Denaturation was performed by incubation of the material in a heat block for 60 min at 55°C, then for 5 min at 105°C to eliminate Proteinase K. DNA was cleaned in Phenol:Chloroform:Isoamylalcohol (25:24:1), then in isopropanol. After 30 min spinning at 14,000 rpm, the obtained pellet was washed twice in 70% Ethanol, then left to dissolve overnight.

The presence of CaMKIICre and MR_flox_ were verified by standardized routine PCR and Southern analysis procedures. Each sample was added in a concentration of 10% to a MasterMix containing 10% PCR buffer, 3% MgCl_2_, 10% dNTP, 2.5% Taq polymerase, 5% of each primer, and distilled water. The following primers were obtained from the Berger lab (Berger et al., 2006):

For MR_flox_:

Primer A (MRflox-10): 5′-CTGAAGTCACTGGCTAGAGTC-3′;

Primer B (MR_flox_-11): 5′-CCAGCCTCTGAGCCCAGAAAG-3′;

Primer C (MR_flox_-12): 5′-GTCCCATCTTGCTTACCCTGA-3′.

For CamKCre:

Primer A (CAMK-13):5′-GGTTCTCCGTTTGCACTCAGGA-3′;

Primer B (CAMK-14): 5′-CCTGTTGTTCAGCTTGCAsCCA-3′;

Primer C (CAMK-15): 5′-CTGCATGCACGGGACAGCTCT-3′.

Amplification consisted of 35 cycles, from 63 to 72°C. After amplification of the target genes, the samples were loaded with 6× loading buffer on 3% agarose gel, and run for 30 min at 100 V. Positive bands at 345 bp for CaMKcre, and 335 bp for MR_flox_ indicated a presence of the genes.

### Early Life Stress Paradigm

ELS was elicited *via* the limited nesting and bedding method, previously adapted to mice by Rice et al. (2008). This model induces chronic stress early in life by means of fragmented and unpredictable maternal behavior, thus affecting the quality but not the quantity of maternal care. Briefly, 1 week before the expected date of birth, dams were individually housed in type II short Macrolon cage with a filter top and provided with a cotton Nestlet (5 × 5 cm, Technilab-BMI, Someren, Netherlands) as nesting material. Around the expected delivery day, cages were checked twice daily for litters. If a new litter was found before 10.00 AM, the previous day was assigned as the date of birth (PND0). Each experimental cage was randomly allocated to either the ELS or control condition. In the morning of P2 (between 09.00 and 10.00 AM), the litters were culled to six pups with approximate equal sex ratio. Litters with pups from the same sex were excluded (*n* = 1). Pups were weighed and housed in either the ELS or control condition in a new polycarbonate type II short cage (268 × 215 × 261 mm, Techniplast™). Control cages were equipped with standard amounts of sawdust bedding and one cotton Nestlet for nesting material (5 × 5 cm; Technilab-BMI, Someren, Netherlands). In the ELS cages, the floor was covered with a little amount of sawdust bedding and was fitted with a fine-gauge stainless steel grid (Naninck et al., 2015). ELS litters were provided with half a piece of Nestlet (2.5 × 5 cm) as nesting material. All cages were covered with a filtertop and left undisturbed for 7 days. On P9, pups were weighed and moved with the dam to standard housing cages. In a pilot [control (*n* = 10) vs. ELS pups (*n* = 11)], body weight gain (4.24 ± 0.19 vs. 1.82 ± 0.18 g; *p* < 0.001), thymus (but not adrenal) weight (0.68 ± 0.03 vs. 0.34 ± 0.05; *p* < 0.001 and 0.03 ± 0.01 vs. 0.03 ± 0.01; *p* = 0.46 for both corrected thymus and adrenal weights) as well as corticosterone levels (3.80 ± 1.14 vs. 13.86 ± 0.93 ng/ml; *p* < 0.001) in the offspring were significantly changed at P9, in accordance with earlier reports (Rice et al., 2008; Naninck et al., 2015). In the remained of the study we used body weight on P9 as a proxy of the effects induced by the model.

After weaning (P21), mice were ear-punched for identification and genotyping, then reallocated and housed per experimental condition in sex-specific rooms with similar housing conditions in type II L cages (365 × 207 × 140 mm, Tecniplast™) until the time of testing in adulthood (10–12 weeks of age). The cohorts that were used for acute restraint stress and protein analysis were socially housed with littermates. The cohort that was used for the behavioral analysis was also group-housed up to the moment that they were individually placed in the home cages i.e., the experimental condition of interest.

### Effect of ELS and MR on Circadian HPA Axis Activity

To assess circadian peak and nadir corticosterone levels, we sampled adult male and female MR_CamKCre/wt_ and MR_flox/wt_ mice (*n* = 11 per condition per sex per genotype), previously exposed to control or ELS condition. Blood samples were collected *via* tail nick, approximately 30–60 min before the light switch (20.00 and 08.00 h). In order to evaluate changes in CORT levels as a measure of circadian HPA axis activity, differences between groups were first analyzed with a 2 × 2 repeated-measures design (for the factor time), in which condition and sex were the independent variables. Next, we tested hypothesis-driven whether MR genotype would exacerbate the findings specifically in the ELS groups which was analyzed using a *t*-test with Holm’s correction. Missing samples were roughly equally distributed over all groups and accounted for 4% in males and 5.5% in females.

To determine corticosterone plasma levels, blood samples were processed as described before (Sarabdjitsingh et al., 2010). Briefly, samples were centrifuged at 13,200 rpm for 10 min at 4°C. Plasma was isolated and stored at −20°C until further use. Plasma CORT levels were determined by using a commercially available radioimmunoassay (RIA) kit (ImmuChemTM Double Antibody, MR Biomedicals, Inc.) according to the manufacturer’s instructions. Samples were counted in duplicate and processed within the same run to avoid inter-assay variation.

### Effect of ELS and MR on Stress-Induced HPA Axis Activity

Acute HPA axis reactivity to restraint stress was expressed absolutely to the baseline blood sample collected the previous day at the nadir when CORT levels are lowest. The next day, mice were placed for 10 min (*t* = 0 starting time) in a cylindrical plexiglass restraint tube (diameter = 6 cm) furnished with breathing holes, then placed solitarily in new cages enriched with bedding material of their old cage to avert novelty-induced stress. For evaluation of the stress-mediated release of CORT, blood samples *via* tail nick were taken at 10-min intervals from stress onset (*t* = 10, *t* = 20, *t* = 30). Blood was collected *via* tail nick in Microvette^®^ tubes (200LH, Sarstedt AG & Co), and never exceeded 20 μL per measurement per animal. The time of first handling of the animal to finish sampling was as fast as possible and never exceeded 2 min. At *t* = 60, the animals were decapitated and trunk blood was collected in tubes (16 I.U. Heparin, Sarstedt AG & Co) containing one granule of heparin to prevent coagulation. HPA axis reactivity was evaluated by testing whether ELS and/or sex affected changes in plasma CORT level over time across groups. Additionally, adrenal glands, thymi and spleens were rapidly dissected and stored at 4°C for a maximum of 3 h before being routinely cleaned and weighted. Body weights of all experimental animals were recorded the day prior to their respective experiment when baseline blood samples were collected. Organ weights were analyzed as a percentage of body weight for each animal.

Results were first analyzed with a 2 × 2 (sex*ELS-condition; repeated measures) ANOVA. If applicable, the secondary analysis was performed with a *t*-test (Holm’s correction for multiple comparisons) for MR genotype in subgroups that were exposed to ELS for each sex separately.

### Effect of ELS and MR on Corticosteroid Receptor Expression in the Brain

The effects of ELS*sex and the potential effect of MR background on MR and GR levels in limbic brain regions were studied by (semi-quantitatively) measuring protein expression with Western blot analysis.

#### Tissue Collection and Sample Preparation

Adult male and female mice (week 10–12) were sacrificed *via* rapid decapitation between 9.30 and 10.30 AM. Dorsal hippocampus (upper third of the hippocampus on the dorsal side) and mPFC of both hemispheres were collected using a stainless-steel brain matrix (RBMA-200C, World Precision Instruments). Hippocampal and mPFC tissues were immediately processed into purified protein, in an RNase-free environment by use of RNaseZAP on all materials (Sigma-Aldrich). Pilot experiments in which we collected amygdala tissue with the use of micro-punches revealed that the amount of tissue collected in this manner was too low to allow reliable Western blot measurements.

As previously described (Sarabdjitsingh et al., 2010; Loi et al., 2015), the samples were homogenized using a homogenizer (IKA^®^ T10 basic) with ice-cold lysis buffer (RIPA) containing 1 M Tris, 1 M NaCl, 0.5% sodium deoxycholate, 0.1% SDS, 1% Triton, and 1 mM EDTA of pH 8. The samples were clarified by centrifugation for 20 min at 13,200 rpm at 4°C, then aliquoted and stored at −80°C for further use.

#### Western Blot Analysis

Protein concentrations were calculated with the slope of a BSA-derived standard curve, by running samples in duplicate with a BCA kit (Pierce, ThermoFisher) in a microplate reader (Varioskan Flash, Thermo Scientific). Approximate equal amounts (15 μg for hippocampal samples, and 17 μg for mPFC) of protein denaturated at 95°C for 5 min were separated on a 10% SDS-polyacrylamide gel (25 mA in stacking gel, 30 mA in resolving gel) and electro-transferred (100 V, 1 h) onto a nitrocellulose membrane (0.45 μm thickness, GE Healthcare Life Sciences, Amsterdam). To reduce unspecific binding, 5% non-fat milk powder (Elk, Campina) in Tris-buffered saline (TBS) was used as blocker for 1 h at room temperature on a shaker. Primary antibody incubation diluted in TBS 1% Tween20 (TBS-T) lasted 3× overnight with mouse monoclonal primary antibody anti-MR (rMR1–18 1D526, dilution 1:500; Gomez-Sanchez et al., 2006), and 1× overnight with rabbit polyclonal antibody anti-GR [GR(M-20), 1:1,000, sc-1004, Santa Cruz Biotechnology Inc., Santa Cruz, CA, USA]. Rabbit polyclonal anti-GAPDH (GAPDH 14C10, 1:3,000, Cell Signalling Technology^®^ Inc., Santa Cruz, CA, USA) was used in both cases as a control protein for standardization. After incubation, membranes were washed 3× for 10 min at room temperature with TBS-T, then incubated with the secondary antibodies diluted in 2% milk TBS-T for 1 h at room temperature. The secondary antibodies used were goat-anti-mouse (1:30,000, Molecular Probes, Eugene, OR, USA) and goat-anti-rabbit (1:50,000) for MR and GR/GAPDH respectively. After washing membranes 3× for 10 min in TBS-T, the proteins were detected using peroxide for chemiluminescent detection of horseradish peroxidase (SuperSignal™ West Dura, Thermo Scientific), and visualized by FluorChemE (proteinsimple™, Westburg). Membranes were then stripped for 5 min at 60°C with stripping buffer composed of 25% Tris, 0.7% β-mercaptoethanol, 2% SDS, and distilled water. After 3 × 5 min washing with TBS, the same procedure from the blocking step was used for the GR protein.

**Figure 1:**
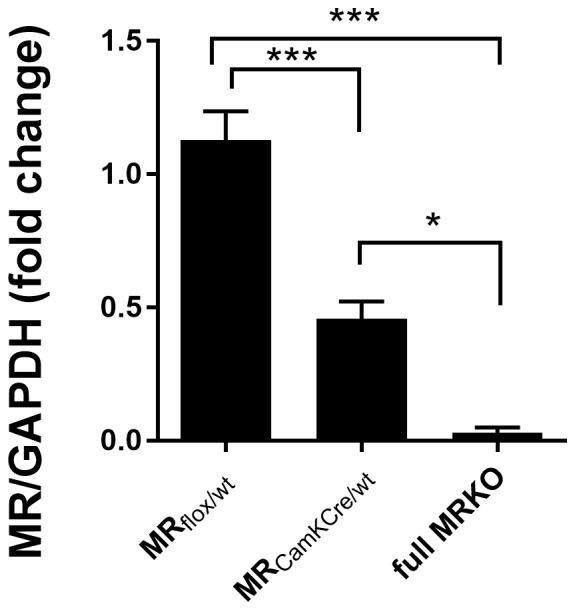
Western blot analysis of mineralocorticoid receptor (MR) expression in the dorsal hippocampus of male MR_flox/wt_ (*n* = 9), MR_CamKCre/wt_ (*n* = 9) and full MRKO mice (*n* = 3). Significant differences across groups, after Holm’s *post hoc* analysis, are indicated: ***p < 0.001, *p < 0.05. Data presented as mean ± SEM.

The loading scheme was randomized, yet each membrane had equal samples per group. In line with previous research, GR and GAPDH were represented on the blots by one line each, respectively at ~90 kDa and ~37 kDa. Conversely, MR displayed two bands at ~130 kDa, thus ~20 kDa heavier than expected. Nevertheless, these bands were confirmed to be the protein of interest as technically validated with samples from full MR knockout mice (Figure 1). The difference in molecular weights is presumably due to posttranslational modifications. The two MR bands were correlated at 0.93; thus only the upper one was quantified. Bands were quantified with ImageJ, corrected for background, standardized with GAPDH, and normalized to MR_flox/wt_ control. Each sample was blotted twice (technical replicates), and the results were then pooled together as mean per animal after normalization.

### Effect of ELS and MR on Novelty-Induced Behavioral Reactivity

We used exposure to a novel environment as a means to study putative changes in stress-induced behavior. We selected novelty-induced behavior because MR is known to modulate the behavioral response to novelty (Berger et al., 2006; Brinks et al., 2009; ter Horst et al., 2012; Arp et al., 2014). A second batch of mice was solitarily placed for a period of 7 h (one time point per hour) in a “home cage,” an automated system for behavior and location tracking, here used to assess novelty-induced activity (Kas et al., 2008; Molenhuis et al., 2014). The home cage apparatus [30 × 30 × 35 (length × width × height)] was built at the University Medical Center Utrecht (NL), and it consists of a home base shelter (10.8 × 6.3 × 6.4 cm), and two *ad libitum* feeding platforms (6.3 × 5.8 × 10.5 cm) of which one is protected and one which is not. The remaining space is furnished with a liter of sawdust and a drinking bottle with tap water (Kas et al., 2008).

Rodent behavior was monitored using the automated video-based observation system PhenoTyper (Noldus Information Technology, Wageningen). The video tracking was performed at a rate of 12.5 samples/s with a spatial resolution of approximately 0.6 mm. Movement was defined as such when start velocity exceeded 2 cm/s and stop velocity exceeded 1.75 cm/s, thus excluding grooming and licking. The data were then extracted with software EthoVision 3.0, which provided mean values per time point requested.

Locomotor activity (proxy for anxiety-like behavior, exploration and strategy) was operationalized as the linear combination of distance moved, time spent moving and velocity. The correlations between these variables did not exceed 0.63. Information about each of these parameters separately is available in our online data files. Due to tracking mistakes, about 1% of values were missing. Additionally, 1.5% of data points were considered as outliers, being outside the 2.5 standard deviation (SD) interval, including in three animals which were outliers in every variable; presumably, these were mostly due to tracking problems. Missing values were randomly distributed across variables and groups.

### Statistical Analysis

Data are presented as mean ± SEM. The analysis was conducted in two stages. First, we tested whether ELS differentially affects males and females in stress-related outcomes by using 2 × 2 (sex*condition) ANOVAs. Second, if *post hoc* tests were significant, we tested whether MR_CamKCre/wt_ genotype enhanced the effects. At this purpose, we compared genotypes in animals with a history of ELS, separately for males and females. This approach was chosen with the intent to limit statistical testing, while still answering primary research questions. Main effects are reported in the text while statistically significant *post hoc* effects are also graphically indicated with symbols.

*Post hoc* analyses were conducted with Holm’s correction for multiple comparisons. Missing values were imputed with a single imputation method, which was repeated five times to verify the sensitivity of the results (van Buuren and Groothuis-Oudshoorn, 2011). Huynh-Feldt correction was used to correct for the departure from sphericity. Concerning AN(C)OVAs’ sum of squares, type III and type II were used, respectively when interaction effects were present or absent. Slopes were calculated as best-fitting. The trapezoid rule was used to calculate the area under the curve. The analysis was conducted in the computer program R (version 3.2.3; Team, 2015), with the aid of the following R packages: (1) “mice” for imputation (van Buuren and Groothuis-Oudshoorn, 2011); and (2) “car” for AN(C)OVAs (Fox and Weisberg, 2011). Statistical significance was assigned at *p* < 0.05.

## Results

### Validation of the Experimental Model

#### MR Expression in the MR_CamKCre/wt_ Is Reduced by ~50%

We used a genetic mouse model to experimentally alter forebrain-specific MR protein levels (Berger et al., 2006). The heterozygous MR mouse is expected to have a 50% reduction of MR protein in limbic brain areas. To test this, MR expression was assessed in a separate batch of male animals (*n* = 9) with western blot analysis in the dorsal hippocampus of adult mice (Figure 1). Compared to MR_flox/wt_ controls, MR expression in the MR_CamKCre/wt_ mice was indeed reduced by 51% (*F*_(2,16)_ = 29, 52; *p* < 0.001; *post hoc* comparison *p* < 0.001). As a reference, MR protein was completely absent in the full knockout MR_CamKCre_ mice. These results confirm that MR expression in the heterozygous mice is indeed reduced in the limbic brain and can be used as a suitable model to study decreased receptor levels in the limbic brain.

#### ELS Effectively Decreases Body Weight Gain at P9 in Both Males and Females

**Figure 2:**
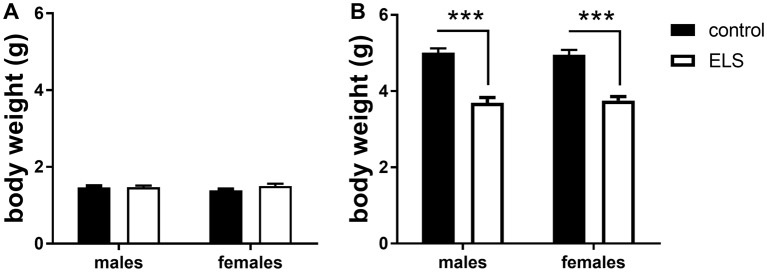
Averaged body weight (±SEM) of the pups in experimental litters **(A)** at the start (P2; **B**) and end (P9) for control (black bars) and early life stress (ELS) condition (open bars). Mean weight per litter was calculated for both males and females. Data were analyzed with averaged litter size at P2 as covariate for the analysis of body weight at P9. Significance indicates main effect of ELS (*F*_(1,44)_ = 89.45, *p* < 0 0.001), ***p < 0.001.

To validate the effectiveness of the ELS paradigm, we monitored body weights of the pups at the beginning (P2) and end (P9) of the experimental condition, a measure commonly affected by stress (Rice et al., 2008; Naninck et al., 2015). At the onset of the model (P2), there were no differences in body weight linked to assigned ELS condition or sex (Figure 2A). A week later at P9, ELS pups had significantly lowered body weight when compared to controls (Figure 2B; main effect of condition *F*_(1,44)_ = 89.45; *p* < 0.001), yet comparable between males and females (main effect of sex *F*_(1,44)_ = 0.03; *p* = 0.87). The MR genotype was unknown at this stage and could therefore not be tested.

#### ELS-Induced Decrease in Body Weight Only Persists in Adult Females

**Figure 3:**
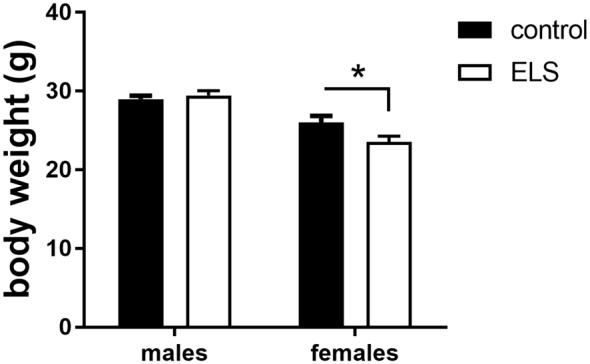
Averaged body weight (±SEM) of adult control (black bars) and ELS animals (open bars). Mean weight per group (±SEM) was calculated for both males and females. Significant differences between groups using a *t*-test with Holm’s correction are indicated, *p < 0.05.

In adulthood, all animals were weighted again to study whether the ELS-induced reduction in body weight would persist (Figure 3). We first analyzed and confirmed a sex*condition effect (*F*_(1,85)_ = 58.3; *p* < 0.05). *Post hoc* analysis showed that the ELS-induced reduction in body weight only persisted in females (*p* < 0.05).

Next, we hypothesized that this ELS-induced reduction in body weight in females was exacerbated by low expression of MR, comparing MR_flox/wt_ to MR_CamKCre/wt_ females after ELS. This difference was however not significantly different (*t*_(13,8)_ = 1.72; *p* = 0.11). All summary statistics are provided in Table 1.

**Table 1:** Summary statistics stratified by sex, condition and genotype for adult body weight, thymus, adrenal and spleen weight (expressed per 100 g body weight).

	MALES				FEMALES			
	MR_flox/wt_		MR_CamKCre/wt_		MR_flox/wt_		MR_CamKCre/wt_	
	Control (11)	ELS (11)	Control (10)	ELS (11)	Control (11)	ELS (11)	Control (13)	ELS (11)
Body weight (g)	29.5 ± 1.06	28.6 ± 2.79	27.7 ± 2.44	30.3 ± 2.17	27.0 ± 4.65	24.8 ± 4.39^#^	25.2 ± 3.86	22.3 ± 1.96^#^
Thymus (mg/100 g BW)	0.139 ± 0.026	0.127 ± 0.023	0.135 ± 0.020	0.119 ± 0.015	0.185 ± 0.061	0.209 ± 0.07	0.229 ± 0.068	0.231 ± 0.035
Adrenals (mg/100 g BW)	0.010 ± 0.003	0.011 ± 0.002	0.012 ± 0.002	0.011 ± 0.003	0.017 ± 0.004	0.02 ± 0.005	0.021 ± 0.008	0.025 ± 0.005
Spleen (mg/100 BW)	0.266 ± 0.049	0.270 ± 0.105	0.262 ± 0.030	0.257 ± 0.035	0.357 ± 0.096	0.383 ± 0.079	0.428 ± 0.177	0.357 ± 0.059

*Values represent group averages (±SEM). ^#^Significant main effect of early life stress (ELS) (t-test using Holm’s correction)*.

### Indices of HPA Axis Under Unstressed, Baseline Conditions in Adulthood

#### Weight of Stress-Sensitive Tissues

We routinely monitored weight of stress sensitive tissues as markers of HPA axis activity, i.e., adrenals, thymus and spleen (Table 1); notably, these values are indicative for both basal and stress-induced conditions. We did not find any significant interaction effect for sex*condition for either thymus (*F*_(1,84)_ = 1.63; *p* = 0.21), adrenal (*F*_(1,84)_ = 2.85; *p* = 0.10) or spleen weight (*F*_(1,84)_ = 0.49; *p* = 0.49).

#### Circadian Variation in Corticosterone Levels

**Figure 4:**
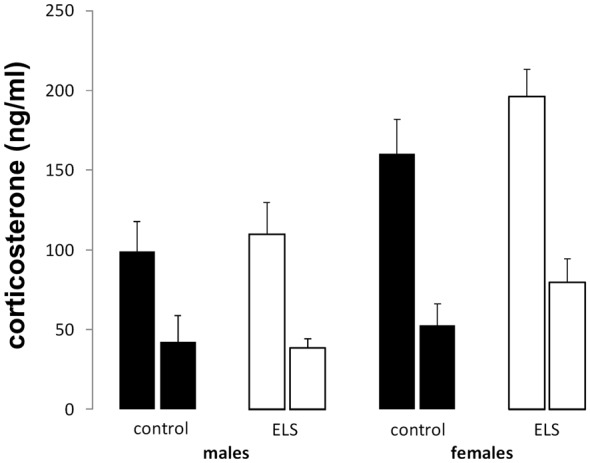
Peak (left bar) and nadir (right bar) plasma corticosterone levels in all experimental groups. Plasma corticosterone was unaffected by ELS (open bars) vs. control (black bars) in both males and females; *n* = 22–24 per group. Data presented as mean ± SEM.

Peak and nadir corticosterone levels were assessed in all experimental groups to study the HPA axis under unstressed condition (Figure 4). All groups displayed typical circadian differences in steroid levels with higher corticosterone concentrations during the peak compared to the nadir (main effect of time *F*_(1,85)_ = 54.74; *p* < 0.001). Additionally, sex-differences in corticosterone levels were also confirmed (main effect of sex (*F*_(1,85)_ = 12.51; *p* < 0.001), with females having higher average circulating corticosterone levels. ELS did not interact with sex (interaction effect: *F*_(1,85)_ = 2.82; *p* = 0.1).

Altogether, these results suggest that none of the indices for baseline (unstressed) HPA axis activity is affected by ELS in either males or females.

### HPA Axis Function Under Acute Stress Conditions in Adulthood

#### Stress-Induced HPA Axis Reactivity

**Figure 5:**
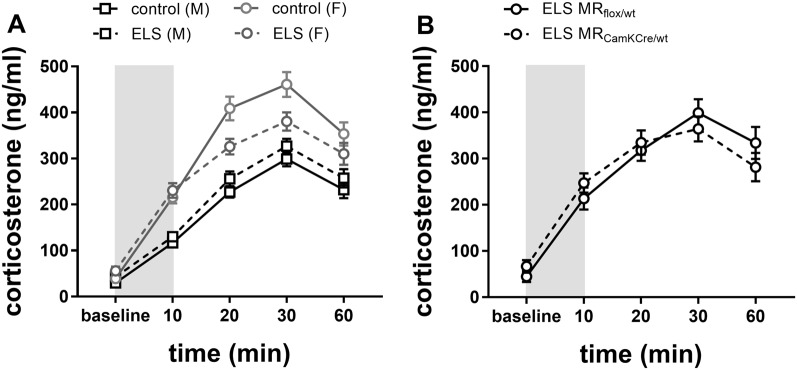
**(A)** Plasma corticosterone levels after immobilization (10 min restraint stress) were analyzed using repeated measures over time in control males and females (black and gray solid line) or in ELS males and females (black and gray dotted line; *n* = 22–24 per group). Data presented as mean ± SEM. **(B)** Plasma corticosterone levels after immobilization plotted for genotype in the females exposed to ELS (*n* = 9–11 per group). Data presented as mean ± SEM.

To investigate HPA axis reactivity to stress, mice were immobilized in a restrainer for 10 min, and plasma corticosterone levels were assessed at different time intervals from onset (Figure 5). We used a repeated-measures analysis to test sex*ELS condition differences in corticosterone levels over time.

Overall, immobilization stress effectively increased corticosterone levels in all experimental groups (Figure 5A; main effect of time *F*_(4,332)_ = 113.73; *p* < 0.001). Females had overall higher averaged corticosterone levels than males (main effect of sex *F*_(1,83)_ = 77.90; *p* < 0.001). As hypothesized, ELS effects had opposing directionality in males compared to females (interaction effect sex*ELS condition *F*_(1,83)_ = 10.64; *p* < 0.01). *Post hoc* analysis indicated that in males a history of ELS marginally increased stress-induced corticosterone levels, although this was only at trend level (*p* = 0.07). Conversely, in females, a history of ELS strongly attenuated the response to restraint stress (Figure 5A; *p* = 0.01).

Next, we tested whether the ELS-induced reduction in stress reactivity in females was exacerbated by MR genotype. *Post hoc* analysis indicated that under ELS conditions in females, the MR_CamKCre/wt_ indeed further decreased corticosterone levels compared to the MR_flox/wt_ controls (Figure 5B; *p* < 0.01).

#### Adult MR and GR Expression

**Figure 6:**
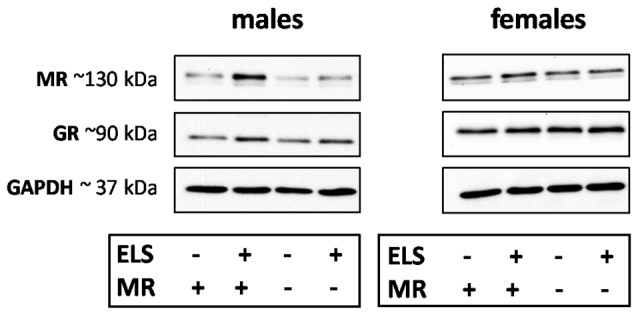
Representative images of MR, glucocorticoid receptor (GR) and GAPDH western blots in male and female samples of the dorsal hippocampus (*n* = 5–9 per experimental group). The results of the medial prefrontal cortex (mPFC) were highly comparable. Legends in the graph: ELS (− or +) refers to either control or ELS condition; MR (− or +) refers to the MR_flox/wt_ or MR_CamKCre/wt_.

We evaluated the effect of ELS and/or MR genotype on MR and GR expression in the dorsal hippocampus and mPFC given their prominent role in the stress circuitry (Jankord and Herman, 2008). All results are described in Table 2. Representative images of the western blot of dorsal hippocampus samples are provided in Figure 6.

**Table 2:** Analysis of Western blot experiments of mineralocorticoid receptor (MR) and glucocorticoid receptor (GR) protein levels in dorsal hippocampus and medial prefrontal cortex (mPFC).

	MALES				FEMALES			
	MR_flox/wt_		MR_CamKCre/wt_		MR_flox/wt_		MR_CamKCre/wt_	
**HC**	**Control**	**ELS**	**Control**	**ELS**	**Control**	**ELS**	**Control**	**ELS**
MR	1.00 ± 0.03 (9)	1.79 ± 0.15 (8)^#^	0.50 ± 0.09 (9)	0.97 ± 0.08 (8)^#@^	1.00 ± 0.09 (6)	1.06 ± 0.14 (5)	0.64 ± 0.07 (6)	0.68 ± 0.08 (6)
GR	1.00 ± 0.02 (8)	1.35 ± 0.08 (8)	1.27 ± 0.09 (9)	1.37 ± 0.11 (8)	1.00 ± 0.04 (6)	1.10 ± 0.06 (6)	1.38 ± 0.12 (6)	1.08 ± 0.05 (6)
**mPFC**	**Control**	**ELS**	**Control**	**ELS**	**Control**	**ELS**	**Control**	**ELS**
MR	1.00 ± 0.07 (9)	1.59 ± 0.20 (8)	0.53 ± 0.08 (9)	0.85 ± 0.14 (8)	1.00 ± 0.16 (6)	0.95 ± 0.19 (6)	0.46 ± 0.03 (6)	0.37 ± 0.07 (6)
GR	1.00 ± 0.09 (9)	1.09 ± 0.11 (8)	0.89 ± 0.07 (9)	0.88 ± 0.06 (8)	1.00 ± 0.07 (6)	1.06 ± 0.05 (6)	1.10 ± 0.10 (6)	0.94 ± 0.03 (6)

*Values indicate normalized group averages (±SEM). ^#^Significant main effect of ELS (t-test using Holm’s correction). ^@^Significant effect of MR genotype in ELS groups: MR_flox/wt_ vs. MR_CamKCre/wt_ (t-test using Holm’s correction)*.

ELS affected MR expression in the dorsal hippocampus in a significantly different manner in males compared to females (interaction effect *F*_(1,55)_ = 5.15; *p* < 0.05). *Post hoc* analysis showed that in males ELS significantly increased MR expression (*p* < 0.01) which was normalized by the MR_CamKCre/wt_genotype (*p* < 0.01). This effect was not found in mPFC (sex*ELS condition effect *F*_(1,56)_ = 0.05; *p* = 0.83). MR expression levels in females were not affected by ELS in either the hippocampus nor mPFC.

Additionally, ELS significantly affected hippocampal GR expression in both sexes (interaction effect sex*ELS condition *F*_(1,55)_ = 4.34; *p* < 0.05). However, *post hoc* analysis showed that only a trend towards increase was observed in males (*p* = 0.07) and not in females (*p* = 0.26). There was no interaction effect in GR expression levels in mPFC (*F*_(1,56)_ = 0.59; *p* = 0.45).

### Behavioral Reactivity to Novelty

We assessed behavioral reactivity to a novel environment (7 h) and place preference for a sheltered space in automated home cages, the latter as a proxy for anxiety.

#### Novelty-Induced Locomotor Activity

**Figure 7:**
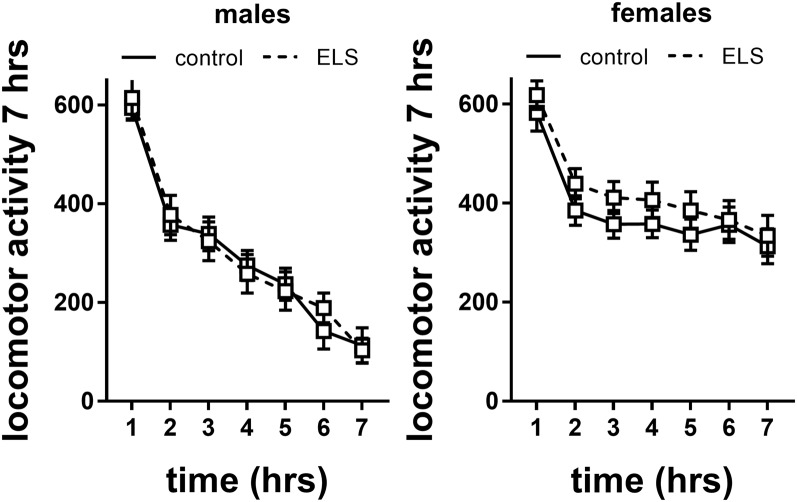
Locomotor activity in males and females exposed to control or ELS condition. Data analyzed with repeated measures ANOVA. *n* = 28–34 per group in males; *n* = 22–26 per group in females. Locomotor activity = linear combination of standardized distance moved, velocity and time spent moving. Data presented as mean ± SEM.

Locomotor activity was operationalized as a composite score of distance moved, velocity and amount of time spent moving, in response to exposure to a novel environment (Figure 7).

Repeated measures analysis revealed that locomotor activity decreased in all experimental groups over time (main effect of time *F*_(6,594)_ = 21.6; *p* < 0.001). This specifically interacted with sex (interaction effect *F*_(6,594)_ = 12.28; *p* < 0.001), with females having higher levels of locomotor activity. This is also reflected in analysis of the slope of the curve (main effect of sex *F*_(1,115)_ = 25.5; *p* < 0.001), suggesting that females did not habituate (albeit we do not have baseline data) to the level of male mice within the tested time frame or adopted different adaptation strategies. There was no interaction of sex with ELS background; therefore, we did not proceed to study the effect of MR genotype.

#### Anxiety-Related Behavior

**Figure 8:**
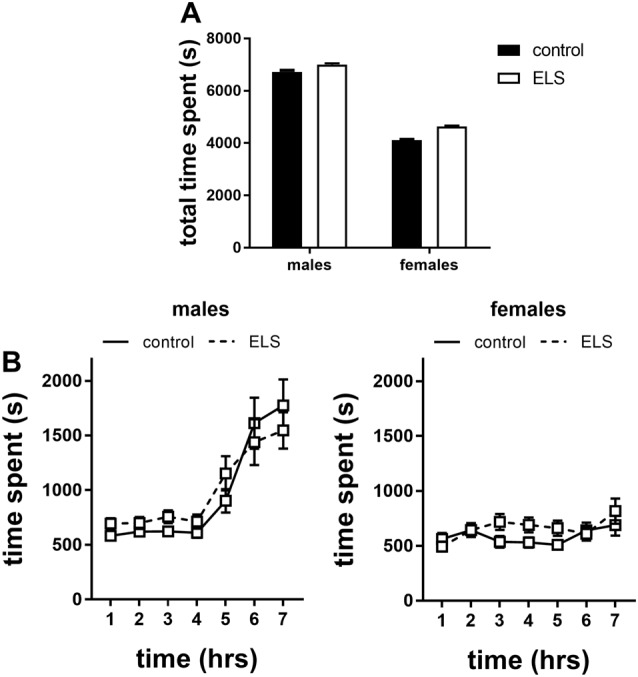
Time spent in the shelter of the home cages in the first 7 h of exposure to a novel environment expressed as **(A)** total time spent and **(B)** over time for males and females exposed to either control or ELS condition (*n* = 18–22 per group in males; *n* = 18–23 per group in females. Data presented as mean ± SEM.

Besides locomotor activity, anxiety-like behavior in a novel environment was also measured as time spent in the shelter (protected area). We found a sex difference in the total time spent in the shelter (Figure 8A; main effect of sex *F*_(1,113)_ = 12.89; *p* < 0.001), with males residing longer in that compartment. No interaction with ELS condition was found.

Interestingly, when the data were expressed over time we found that this sex difference appeared after the 4th hour in the novel environment in which males increased their time in the shelter (Figure 8B; interaction effect of time*sex *F*_(6,642)_ = 13.13, *p* < 0.001); this actually challenges the interpretation of time spent in the shelter as a proxy for anxiety (see “Discussion” section).

Altogether, these data suggest that males and females gradually respond behaviorally differently to novel, mildly stressful situations, by adopting different coping strategies. This was not influenced by ELS.

## Discussion

Aberrant regulation of the HPA axis has been suggested to contribute to vulnerable phenotypes in stress-related psychopathology (de Kloet et al., 2005; Bale, 2006). This is supposedly caused by an interplay between genetic predisposition and environmental challenges (Claessens et al., 2011; Daskalakis et al., 2013), of which the sequence of events is still largely unknown. To follow this in a prospective manner, the current study aimed to tie together recently identified factors in psychopathology and the mediating role of HPA axis functioning: (i) sex-dependent effects of ELS; and (ii) a putative modulation by the MR (Heim et al., 2008; Strüber et al., 2014; ter Heegde et al., 2015; Joëls and de Kloet, 2017). We specifically focused on the role of the MR, in view of recent evidence supporting that some MR haplotypes confer resilience to depression in a sex-dependent manner (Vinkers et al., 2015); this does not negate that variants of the GR gene may also contribute to the vulnerability to develop psychopathology in the face of early life events (e.g., Sarubin et al., 2017).

We report four main findings: (i) indices of HPA axis activity under non-stressed conditions are different between males and females but are not affected by ELS; (ii) immobilization stress induced opposing ELS-specific effects, with increased corticosterone levels in males but decreased in females. In females, low MR expression levels exacerbated this effect; (iii) in males but not females, ELS increased MR expression in dorsal hippocampus and mPFC in male mice; and (iv) when exposed to a novel environment, locomotor activity was increased in both male and female mice, with females adopting a different adaptation strategy than males.

### Validity of the Model

In the current study, the limited nesting and bedding model was used as an environmental challenge to mimic adverse early life conditions in the human situation. This model was originally developed in the Baram lab as a translational tool to investigate the neurobiological underpinnings of early life programming (Brunson, 2005; Rice et al., 2008), in an effort to model the aberrant patterns in maternal behavior often seen in mentally ill or abusive mothers (Walker et al., 2017). Such patterns in maternal-derived signals influence brain circuit maturation important for memory, cognitive and affective functioning thereby promoting resilience or vulnerability to mental illness (Glynn and Baram, 2019).

The limited nesting and bedding model was applied to mice carrying the heterozygous knockout of the MR gene *Nr3C2*, to model the vulnerable genetic phenotype of the MR haplotypes that are linked to stress-related psychopathologies (ter Heegde et al., 2015). We confirmed a 50% reduction in MR expression levels in the hippocampus and tentatively conclude that this animal model is suitable for studying differential expression of MR in the brain with good external validity and generalizability to human findings. It should be noted, however, that the functionality and underlying mechanisms of the MR haplotypes have not yet been extensively investigated (van Leeuwen et al., 2011). We, therefore, cannot exclude the influence of changes additional to the MR expression level (e.g., ligand binding and all functionality involving other proteins) or potential interference of a slight upregulation of GR (Berger et al., 2006). Additionally, although the *Nr3C2* gene encodes both nuclear and membrane-bound MR, to the best of our knowledge it is not possible with the current techniques to verify whether the observed 50% reduction is proportional in both types. Given the different roles of cytoplasmic and membrane-bound MR (Joëls et al., 2012), this assumption might influence the interpretation of the data. Finally, while Berger et al. (2006) reported that the MR_CamKCre_ is reduced at P0 and almost lost at P6, we did not test whether the reduction in MR expression was indeed present at the onset of the ELS paradigm.

### HPA Axis Reactivity to Stress

HPA axis functionality might contribute to variations in disease susceptibility after ELS (Nestler et al., 2002; Heim et al., 2008; de Kloet et al., 2016). The neural circuitry of the HPA axis is highly evolutionary conserved and therefore provides a strong translational measure. Indeed, dysregulation of HPA axis activity is apparent in rodent studies of ELS albeit sometimes with conflicting results (Wang et al., 2012; Molet et al., 2014; McIlwrick et al., 2016; van Bodegom et al., 2017). In our study, indices for HPA axis activity under rest were not affected by ELS, which suggests that ELS-induced changes in the stress system did not impair homeostasis and consequently confound other results. Yet, we cannot exclude changes in HPA axis activity early in life as we did not assess corticosterone levels shortly after the stress paradigm. Evidence from other studies, in fact, point to an early onset of HPA axis dysregulation originating during the stress hyporesponsive period (SHRP; P2–12) with lasting consequences of neuroendocrine programming into adulthood, similar to our study (Sapolsky and Meaney, 1986; McIlwrick et al., 2017).

In line with our hypothesis, we observed striking, opposing sex-dependent ELS differences in the directionality of the transient stress response evoked by a brief period of restraint stress: decreased CORT levels in females and—at trend level—increased levels in males. This is in line with a recent meta-analysis in humans showing sex-differences in HPA axis reactivity related to depression (Zorn et al., 2017).

### Role of MR

We considered the possibility that hippocampal and prefrontal MR (or GR) levels may contribute to the sex-dependent changes in HPA axis reactivity. We report that the limited nesting and bedding model particularly affected MR expression in males. A search of the existing literature resulted in seven articles that investigated ELS-mediated changes of MR expression in the hippocampus, but none for the PFC. They either describe a downregulation (Maccari et al., 1995; Barbazanges et al., 1996), upregulation (Ellenbroek and Cools, 2000; Nasca et al., 2015; Marasco et al., 2016) or no change in MR expression (Wang et al., 2012; van der Doelen et al., 2014). These studies were only conducted in males, investigated mRNAs, differed in the technique used, the exact part of the hippocampus investigated, the ELS model adopted and its timing (pre- or post-natal) which hampers the comparison. Interestingly, among the abovementioned studies, both highly susceptible rats (APOSUS) and mice are characterized by increased MR expression in the hippocampus (Ellenbroek and Cools, 2000; Nasca et al., 2015). Concerning APOSUS rats, it was concluded that the vulnerability was enhanced by maternal behavior since cross-fostering ameliorated the phenotype (Ellenbroek and Cools, 2000). We argue that these studies may best resemble the limited nesting model used here as their hallmark is altered quality and not the quantity of maternal behavior (Rice et al., 2008; Glynn and Baram, 2019).

If the upregulation of limbic MR after ELS in males—and no effect in females—would be the main driver of the changes in stress-reactivity, we would expect to see a *lower* threshold and peak of stress-induced corticosterone levels in ELS males and no change in females; this assumption is based on the inhibitory role of (at least hippocampal) MR on the HPA axis (Joëls and de Kloet, 2017). However, this is not what we observed. One explanation is that the ELS-induced changes in corticosterone response to stress occurs independent of hippocampal MR expression. Rather, the ELS-induced MR increase in males may mitigate the effects of ELS on the corticosterone response, resulting in a relatively modest change in HPA-reactivity. In females there is no such compensatory effect by MR expression, resulting in a stronger phenotype of the stress-induced corticosterone release. However, the overall influence of brain MR on HPA axis reactivity is not only determined by the two areas we presently investigated. For instance, the amygdala exerts a stimulatory role on stress-induced corticosterone release (Ulrich-Lai and Herman, 2009). We cannot exclude that the balance between MR changes in such stimulatory areas outweighs the change in inhibitory regions. This would agree with the observation that reduction of MR expression in (all) CamKII-expressing cells in the forebrain exacerbates the ELS-induced changes in corticosterone release. Finally, the effect of MR is closely linked to GR-dependent processes. Although we did not observe any sex*ELS interaction, the fact that ELS did change GR levels may have consequences for the stress-induced CORT release.

Why male and female mice respond differently to ELS is not explained by our study. The neurobiological mechanisms by which ELS affects stress responsivity are complex. In males, this is most likely due to reduced inhibitory drive to the glutamatergic innervations of the CRH neurons in the PVN, resulting in elevation of corticosterone levels (Gunn et al., 2013). Yet, the rodent females’ literature is limited. Just a few studies addressed this issue experimentally although comparability is hampered due to differences in methodology, with respect to intensity and duration of the stressor, genotype and statistical analysis (Machado et al., 2013; van Bodegom et al., 2017). A possible explanation for the observed ELS-induced hyporeactivity in females comes from rodent experiments on the ultradian rhythm, which suggested that following ELS there is an increase in the frequency of the ultradian pulses, similar to chronic stress (Windle et al., 2008). This results in an increased refractory time in which the animals are in a non-responsive state, giving rise to apparent stress hyporesponsiveness (Windle et al., 2008; Lightman and Conway-Campbell, 2010; Sarabdjitsingh et al., 2010). From a mechanistic perspective, males’ and females’ differences in MR expression can also be explained by considering influences of progesterone and estrogen on MR expression (Carey et al., 1995; Quinkler et al., 2002; Barrett Mueller et al., 2014). Clearly, dedicated neuroendocrine experiments in female rodents are needed to delineate the exact underlying mechanisms investigating the sex-dependency in the observed phenomena.

### Behavioral Reactivity to Stress

Many studies support that exposure to stress during sensitive periods in life can contribute to severe, long-lasting behavioral consequences in affective disorders (Eiland and McEwen, 2012; Pagliaccio et al., 2015; Fonzo et al., 2016). Although little is known how adverse experiences in early life may-interact with the sexually dimorphic programming of the brain, sex differences are apparent (McCauley et al., 1997; Bale and Epperson, 2015).

A vast majority of rodent studies also support that ELS induces changes in behavioral indicators of anxiety later in life (Schmidt et al., 2010; Hartmann et al., 2012; Wang et al., 2012; Cotella et al., 2014; Pan et al., 2014; Sachs et al., 2015; Kanatsou et al., 2016). This is most likely due to ELS-induced accelerated maturation of the fear circuitry, possibly resulting in hyperactivity of the amygdala (Raineki et al., 2012; Bath et al., 2016). In the current study, we tracked behavioral characteristics in a novel environment. We did not find any effect of ELS on anxiety-related behavior although males and females displayed a difference in their adaptation curve. Possibly, (1) females do not adapt to the environment, or (2) females have an enhanced basal activity and minimally respond to a novel environment, therefore reach their baseline sooner. Additional experiments would be required to investigate this.

We selected behavioral reactivity to a novel situation as output parameter, because MR has been in implicated in the adaptation to stress and the appraisal of novel situations in both rodents and humans (Ferguson and Sapolsky, 2007; Schwabe et al., 2013; Arp et al., 2014; Vogel et al., 2016). Specifically, female MR deficient mice seem to be more anxious and lack the behavioral flexibility that is required to rapidly adapt to novel settings and choose appropriate coping strategies in stressful situations (ter Horst et al., 2012, 2013; Joëls and de Kloet, 2017). Conversely, previous studies have argued that overexpression of MR may have anxiolytic effects (Rozeboom et al., 2007; Mitra et al., 2009), albeit not in the context of ELS (Kanatsou et al., 2016, 2017).

## Conclusion

Recent human studies reported sex-dependent differences in stress-reactivity, a factor that is known to be sensitive to early life environment and thought to be involved in individuals’ susceptibility to depression. This also depends on genetic background e.g., the MR haplotype (Vinkers et al., 2015): In males, the risk to develop depression after childhood maltreatment was dampened if the individuals carried MR haplotypes thought to result in low expression. By contrast, these haplotypes resulted in increased vulnerability in females. However, the complex interplay between sex, early life environment and MR background on HPA axis reactivity have not yet been tested in a comprehensive, prospective design. To do so, we reverted to a well-controlled animal study. Our results indeed support that ELS alters HPA axis functioning sex-dependently and that reduction of MR levels exacerbates this pattern in females.

## Data Availability

The raw data supporting the conclusions of this manuscript will be made available by the authors, without undue reservation, to any qualified researcher.

## Ethics Statement

This study adheres to all above requirements. The current study was approved by the Animal Ethical Committee from Utrecht University, Netherlands. Every effort was taken to minimize animal suffering in accordance with the FELASA guidelines and the Dutch regulation for housing and care of laboratory animals (January 30th 2001/GZB/VVB 2148400).

## Author Contributions

VB, MA, RD and RS carried out the experiment. VB, RS and MJ conceived the original idea and wrote the manuscript. VB, MA and RS analyzed the data. LW and OM contributed technically to the experiment. RS supervised the project.

## Conflict of Interest Statement

The authors declare that the research was conducted in the absence of any commercial or financial relationships that could be construed as a potential conflict of interest.
